# Novel Methodology for Creating Macaque Retinas with Sortable Photoreceptors and Ganglion Cells

**DOI:** 10.3389/fnins.2016.00551

**Published:** 2016-12-01

**Authors:** Shreyasi Choudhury, Christianne E. Strang, John J. Alexander, Miranda L. Scalabrino, Julie Lynch Hill, Daniel T. Kasuga, C. Douglas Witherspoon, Sanford L. Boye, Paul D. Gamlin, Shannon E. Boye

**Affiliations:** ^1^Department of Ophthalmology, University of FloridaGainesville, FL, USA; ^2^Department of Psychology, University of Alabama at BirminghamBirmingham, AL, USA; ^3^Department of Human Genetics, Emory UniversityAtlanta, GA, USA; ^4^Department of Ophthalmology, University of Alabama at BirminghamBirmingham, AL, USA

**Keywords:** macaque, photoreceptors (PRs), retinal ganglion cells (RGCs), adeno associated virus (AAV), subretinal injection, lateral geniculate nuclei (LGN) injection, fluorescent activated cell sorting (FACS)

## Abstract

**Purpose:** The ability to generate macaque retinas with sortable cell populations would be of great benefit to both basic and translational studies of the primate retina. The purpose of our study was therefore to develop methods to achieve this goal by selectively labeling, in life, photoreceptors (PRs) and retinal ganglion cells (RGCs) with separate fluorescent markers.

**Methods:** Labeling of macaque (*Macaca fascicularis*) PRs and RGCs was accomplished by subretinal delivery of AAV5-hGRK1-GFP, and retrograde transport of micro-ruby™ from the lateral geniculate nucleus, respectively. Retinas were anatomically separated into different regions. Dissociation conditions were optimized, and cells from each region underwent fluorescent activated cell sorting (FACS). Expression of retinal cell type- specific genes was assessed by quantitative real-time PCR to characterize isolated cell populations.

**Results:** We show that macaque PRs and RGCs can be simultaneously labeled in-life and enriched populations isolated by FACS. Recovery from different retinal regions indicated efficient isolation/enrichment for PRs and RGCs, with the macula being particularly amendable to this technique.

**Conclusions:** The methods and materials presented here allow for the identification of novel reagents designed to target RGCs and/or photoreceptors in a species that is phylogenetically and anatomically similar to human. These techniques will enable screening of intravitreally-delivered AAV capsid libraries for variants with increased tropism for PRs and/or RGCs and the evaluation of vector tropism and/or cellular promoter activity of gene therapy vectors in a clinically relevant species.

## Introduction

The ability to manipulate gene expression within the retina aids in our understanding of the molecular mechanisms underlying degenerative retinal disease. Furthermore, targeted gene expression in specific cell types offers the possibility of modeling human disease and developing treatment strategies. From a utilitarian perspective, it can be also used to selectively label retinal cells via the differential expression of fluorescent proteins. These cells can then be isolated by fluorescence activated cell sorting (FACS) for further characterization (Chan et al., 2004; Morgan et al., 2005; Akimoto et al., 2006; MacLaren et al., 2006; Dhingra et al., 2008; Lakowski et al., 2010; Pearson et al., 2012; Mansergh et al., 2015). In mice, manipulations of genes in specific retinal cell types can be achieved via traditional germline transgenesis. However, while transgenic mice are invaluable for many studies, their utility as a model for human disease is limited by substantial differences in their ocular anatomy/retinal topography relative to primates. These differences include, but are not limited to, the absence of a cone exclusive fovea (the region of the primate retina responsible for high acuity vision), differential patterns of retinal vascular and basement membrane thicknesses and a significantly smaller globe. These differences present challenges for translating findings in mice to patients with retinal disease and point to the need to develop useful models in primates. Macaques, (genus *Macaca*), have ocular characteristics most similar to man (Curcio and Allen, 1990; Rolling, 2004; Frenkel et al., 2005; Buch et al., 2008; Stieger et al., 2009; Beltran et al., 2010) but, due to cost and time, germline transgenesis is currently not feasible. Therefore, a different approach is necessary.

Adeno associated virus (AAV)-mediated transgenesis is a frequently used method for modifying gene expression in specific retinal cell types after terminal differentiation and has been employed to create various rodent models of human retinal disease (Matsumoto et al., 1984; Qi et al., 2003; Justilien et al., 2007; Yu et al., 2012). Conversely, AAV-mediated gene supplementation has been used to correct phenotypes in a myriad of retinal disease models (Boye et al., 2013; Carvalho and Vandenberghe, 2015; Schön et al., 2015). The establishment of AAV serotype and promoter combinations capable of restricting transgene expression to photoreceptors has been further extended to non-human primates (NHP; Vandenberghe et al., 2011, 2013; Boye et al., 2012; Ramachandran et al., 2016) and can be used in conjunction with a separate method that fluorescently labels retinal ganglion cells (RGCs). RGC axons project to various regions of the primate brain with the largest projection to the lateral geniculate nucleus (LGN). Fluorescent dextrans injected into the LGN of macaques undergo retrograde transport to the cell bodies of ganglion cells, thus selectively labeling these cells (Dacey et al., 2003, 2005). By combining both AAV-mediated transgenesis of PRs and retrograde labeling of RGCs, the same type of experimentation carried out in mouse models with constitutively fluorescing retinal cells is now possible in macaques.

In this report we describe a combinatorial approach to create, in life, macaque retinas containing both sortable photoreceptors and RGCs, and methods for efficiently isolating and validating each respective cell population. This general method may be used to interrogate transduction profiles of existing vectors as well as the activity of regulatory elements used for driving transgene expression. As has been previously done with transgenic reporter mouse models (Kay et al., 2013; Cronin et al., 2014; Boye et al., 2016), this approach can also be used as a screening method for identifying novel viral vectors (i.e., within capsid libraries) best suited for targeting individual retinal cell populations in a clinically relevant species.

## Materials and methods

### Animals

Two adult, male macaques (*Macaca fascicularis*) were used in this study. All procedures performed on macaques were approved by Institutional Animal Care and Use Committees at the University of Alabama at Birmingham (UAB) and performed in accordance with the Association for Research in Vision and Ophthalmology Statement for the use of animals in ophthalmic and vision research. Animal AV263 (Age ~20 years) received no in-life treatment. Retinal cells were labeled with micro-ruby™ post sacrifice. Animal SA76A (Age ~8 years) received in-life bilateral subretinal injections of AAV to label photoreceptors and bilateral LGN injections of micro-ruby™ to label RGCs (Tables 1, 2).

**Table 1:** **Animal and experimental details**.

**ID#**	**DOB**	**Photoreceptor Labeling**		**RGC Labeling**		**Sacrifice**
		**Label**	**Method**	**Label**	**Method**	
AV263	8/17/1995	PNA- 5 μg/ml (1 mg/ml diluted 1:20) in 0.1 M PBS/5% FBS	Dissociated cells incubated with 5 μg/ml PNA for 15 min at RT	Microruby-2.5 mg/ml diluted in oxygenated Ames media	Optic nerve head incubated with 2.5 mg/ml microruby solution for 3 h at RT	4/12/2015

**Table 2:** **Animal and experimental details**.

**ID#**	**DOB**	**Photoreceptor Labeling**		**RGC Labeling**		**In life imaging**	**Sacrifice**
		**Injection date**	**Vector, volume**	**Injection date**	**Volume**		
SA76A	6/8/2007	5/2/2015	AAV5-hGRK1-GFP	5/27/2015	Microruby injections	5/22/2015	6/3/2015
			1 × 10^12^		at 6–8 sites in LGN		
			OS- 480 μl injected		in both hemispheres		
			at 5 sites		Total volume per LGN = 6 μl		
			OD- 250 μl injected				
			at 5 sites				

### AAV vector

The AAV vector comprising the 292 base pair human rhodopsin kinase promoter (hGRK1) driving GFP (Beltran et al., 2010) packaged in AAV serotype 5 has been previously described (Zolotukhin, 2005; Jacobson et al., 2006a). Virus stock at a titer of 8.0 × 10^13^ vector genomes per milliliter (vg/ml) was diluted in the same buffer used for AAV storage, balanced salt solution (BSS) (Alcon, Fort Worth, TX) with 0.014% Tween 20 (JT Baker, Philipsburg, NJ) to the delivered dose concentration of 1 × 10^12^ vg/ml ~1 h prior to subretinal injections. The vector dosing solution was allowed to warm to room temperature to prevent off-gassing during injection.

### Micro-ruby preparation

Tetramethylrhodamine and biotinylated dextran 3000 MW, lysine fixable (micro-ruby™, #D-7162; Thermo Fisher Scientific) was reconstituted to 10% in sterile saline.

### Subretinal injections

Subretinal injections in macaque eyes were performed according to our previously published methods (Boye et al., 2012). For a more detailed description, see Supplementary Materials.

### *In vivo* imaging

In-life GFP expression was observed 20 days post-injection using a Topcon TRC 50EX fundus camera equipped with a Fundus Photo adapter, Canon EOS 6D digital camera, and New Vision Ophthalmic Imaging Software (Fundus Photo, LLC). This system employs interchangeable, custom excitation and barrier filters, and images of GFP expression were captured with a 469 nm excitation filter (Semrock FF01-469/35-32-D) combined with a 525 nm barrier filter (Semrock FF02—525/40).

### Lateral geniculate nuclei (LGN) injections

Twenty five days post-subretinal injection and 5 days post- fundus imaging, the lateral geniculate nuclei of animal SA76A were injected bilaterally with micro-ruby™ to retrogradely label RGCs as described previously (Dacey et al., 2003, 2005). For a more detailed description, see Supplementary Materials.

### Tissue processing

**Figure 1:**
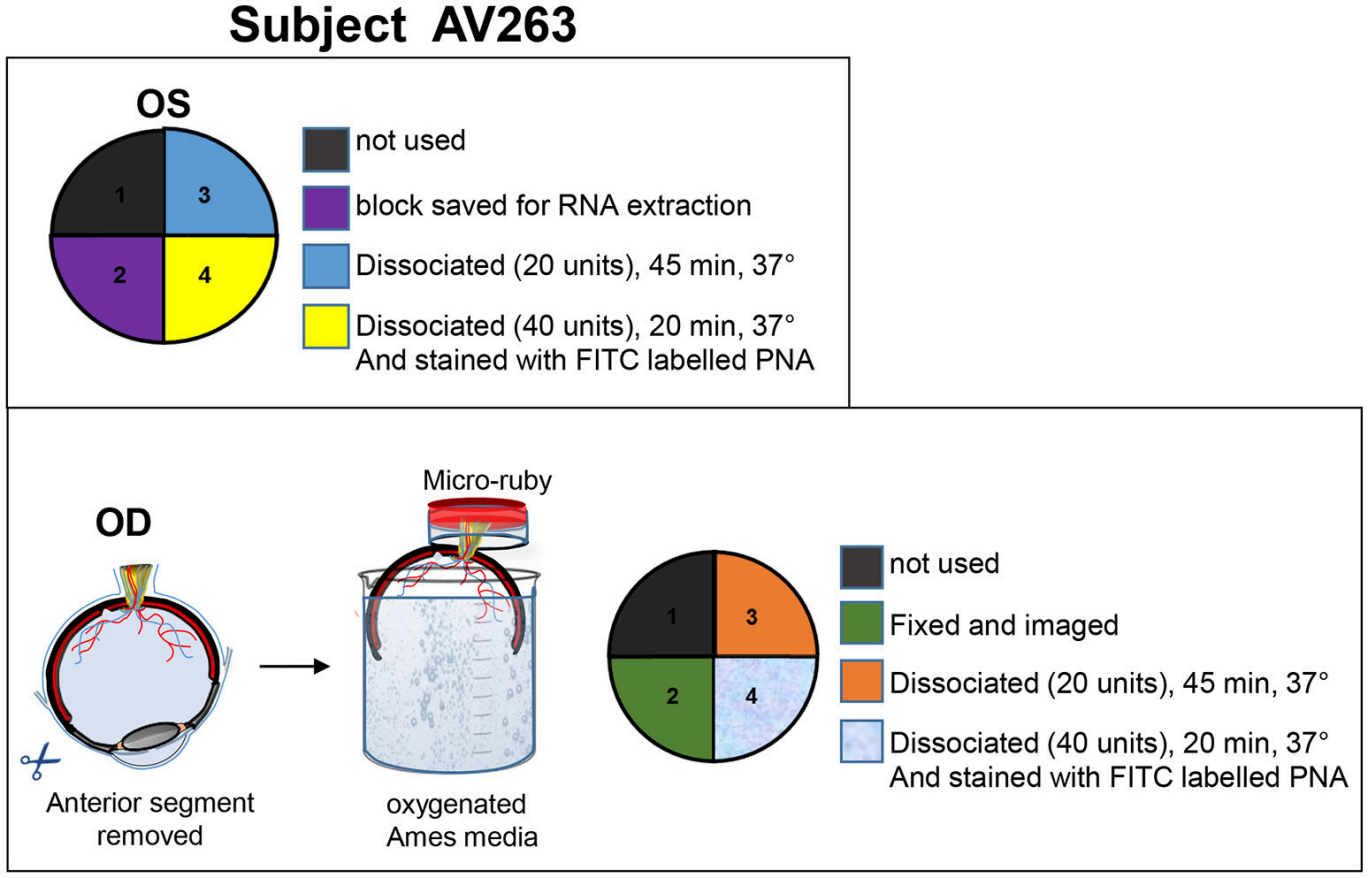
**Details of tissue processing from OD and OS eyes of animal AV263**. Anterior segment and vitreous were removed from both eyes and eyecups immersed in oxygenated Ames media. Retinas were carefully isolated from RPE. The OS retina was divided into four quadrants, each intended for purposes described within the **top panel**. During incubation in Ames media, optic nerve from the OD eyecup was incubated in 2.5 mg/mL micro-ruby™ diluted in oxygenated Ames media for 3 h at room temperature. The OD retina was then divided into four quadrants, each intended for purposes described within the **bottom panel**.

At the time of sacrifice (32 days post-subretinal injections for SA76A), animals were deeply anesthetized and eyes were enucleated. The animals were euthanized immediately after enucleation. The anterior chamber and the vitreous were removed. The resulting eyecup was immersed in oxygenated Ames media while the retina was carefully and thoroughly isolated from the retinal pigment epithelia (RPE). Retina from the OS eye of animal AV263 was then dissected into four quadrants (Figure 1). Retinal tissue from quadrant 1 was set aside for use in an unrelated experiment. Quadrant 2 tissue was immersed in RNA later solution (Qiagen, CA, Cat #76104). RNA was extracted and cDNA prepared from this quadrant to validate macaque-specific primers to retinal expressed genes. Retinal tissue from quadrants 3 and 4 were used to optimize cell sorting conditions. Animal AV263's OD eye was hemisected and submerged into oxygenated media as described above. To retrogradely label RGCs, the eye was stabilized with the optic nerve end up, a small piece of tygon tubing was attached to the back of the eye cup, creating a well around the severed end of the optic nerve. 2.5 mg/mL micro-ruby diluted in oxygenated Ames media was added to the well and allowed to incubate for 3 h at room temperature, after which the eye was dissected (maintaining orientation) and the retina cut into 4 quadrants. Quadrant 1 (superior nasal retina) was set aside for use in an unrelated experiment. Retina from quadrant 2 (inferior nasal) was fixed for 1 h at room temperature in 4% PFA, washed and mounted for imaging (data not shown). Retinal tissue from quadrants 3 (superior temporal) and 4 (inferior temporal) were used to optimize cell sorting conditions as described below. The dissociated cells from quadrants 3 and 4 were incubated with 5 μg/ml Alexa-488 fluorophore-conjugated peanut agglutinin (PNA) in 1x PBS/5% FBS for 15 min at room temperature. A detailed summary of experimental design for animal AV263 can be found in Figure 1.

**Figure 2:**
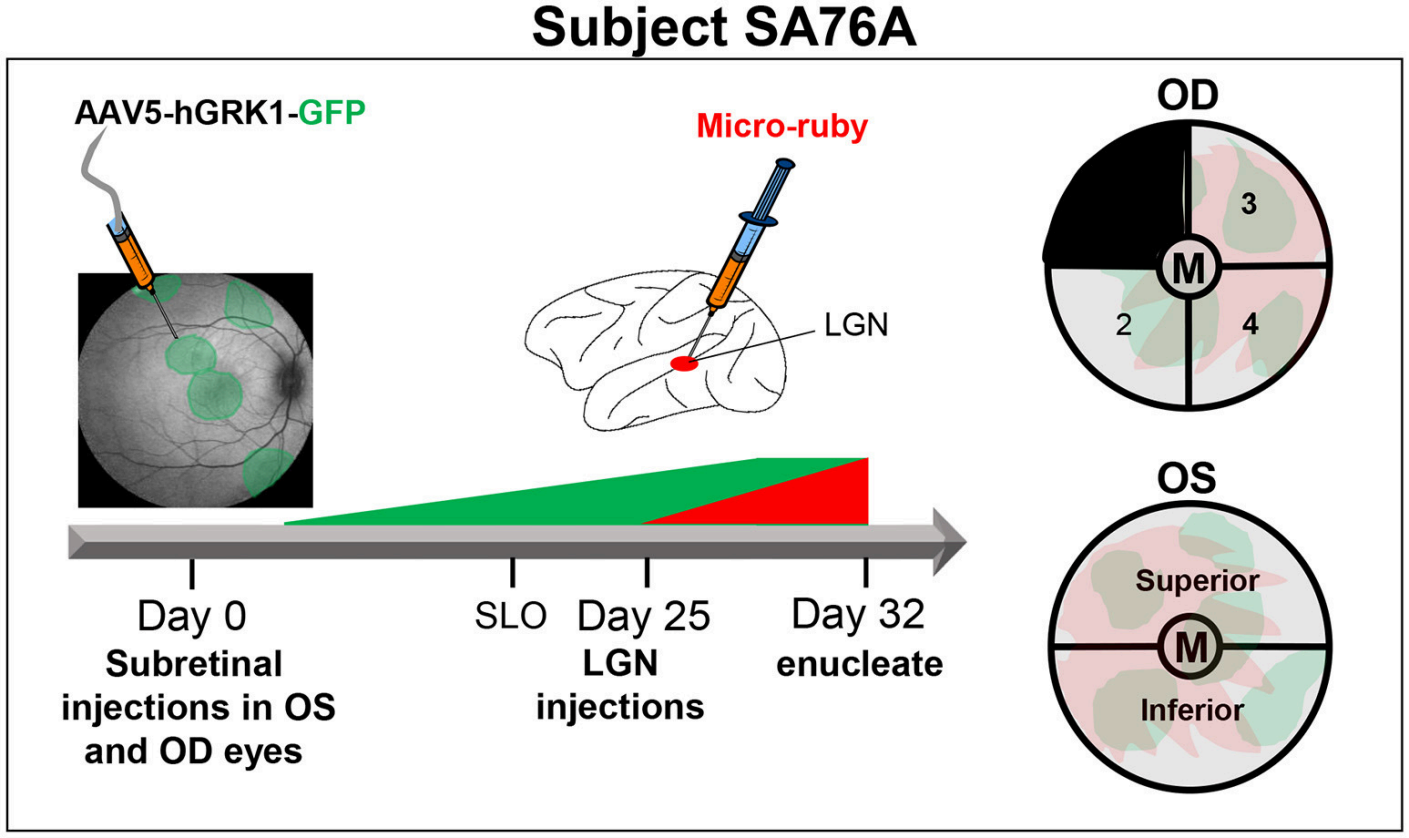
**Experimental design and details of tissue processing from OD and OS eyes of animal SA76A**. Subretinal injections of AAV5-hGRK1-GFP (1.0 × 10^12^ vg/ml) were performed in five different sites of both OD (480 μl total) and OS (250 μl total) eyes. At 20 days post-injection, in life images (fluorescence fundus) were taken to confirm GFP expression within the subretinal blebs. Three days later, injections of micro-ruby™ were performed at a total of 9 sites (0.6 μl per site) within both lateral geniculate nuclei (LGN). At 32 days post injection, eyes were enucleated, and anterior segment and vitreous were removed. Four millimeters punches were made to isolate the macula/fovea from both eyes. The remaining OS retina was divided into superior and inferior hemispheres. The remaining OD retina was divided into 4 quadrants. Quadrant 2 of the OD eye was fixed and mounted for imaging. Quadrant 3- OD was combined with superior OS retina. Quandrant 4- OD was combined with inferior OS retina. The macula/fovea punches from both eyes were combined. All pooled samples were subsequently dissociated and sorted.

From the eyecups of animal SA76A, 4 mm punches were made with sterile disposable biopsy punch (Sklar surgical instrument, PA Cat #SK96-1115) to isolate the macula/fovea under a dissecting microscope. The remaining OS retina was divided into superior and inferior hemispheres. The OD retina minus the macula was divided into four equal quadrants. Quadrant 1 (temporal superior retina) was kept for an unrelated experiment. Quadrant 2 (temporal inferior retina) was fixed for 1 h at room temperature in 4% PFA and mounted for imaging with a Zeiss Axioscope wide field fluorescence microscope with a 40 × 1.4 NA objective. Quadrants 3 and 4 (nasal inferior and nasal superior retina, respectively) were combined with the inferior and superior regions from the OS retina, respectively to constitute the “Superior” and “Inferior” samples. The macula/foveal punches from both OD and OS eyes were combined to constitute the “Macula/Fovea” sample. Samples were dissociated and analyzed by FACS. A detailed summary of experimental design for animal SA76A can be found in Figure 2.

SA76A was perfused through the aorta with 2 L 1% Sodium nitrite/0.9% Sodium chloride, and fixed with 4L of 4% paraformaldehyde in 0.1 M phosphate buffer. The brain was stereotaxically blocked in the coronal plane at the level of the brainstem, placed in 30% sucrose in 0.1 M phosphate buffer for 3–5 days, and then sectioned at 40 μm on a freezing, sliding microtome (AO 860). All sections from the level of the optic chiasm through the posterior LGN were collected for later processing. Selected sections though the LGN were rinsed and incubated in streptavidin-HRP (Rockland, 1:1000) in PBS plus 0.5% triton X-100. They were then rinsed and reacted with 3′, 3′ diaminobenzidine (DAB), Pierce metal-enhanced solution for 12 min. Sections were rinsed, mounted on gel-subbed slides, and allowed to dry. They were then defatted in Xylene, and coverslipped with Permount. Whole brain sections were imaged at 2.65 μm resolution on a Canon 9000F mark II slide scanner. Contrast and brightness of images were subsequently adjusted in Photoshop for optimal clarity.

### Retinal dissociation and FACS

The various retinal samples from animals AV263 and SA76A were dissociated with papain (Worthington Biochemical Corporation, NJ, Cat #3150) according to the manufacture's protocol. In brief, papain was pre-incubated in 5 ml of Earle's Balanced Salt Solution (EBSS) for 10 min at 37°C. After pre-incubation, 250 μl of DNase (dissolved in either 500 μl or 250 μl of EBSS) was added to a final concentration of ~20 or 40 units/ml papain and 0.005% DNase. Dissected retina samples were placed in 15 ml falcon tubes containing 700 μl of papain/DNase and equilibrated with 95% O_2_:5% CO_2._ Retina blocks were dissociated by incubation with activated papain at 37°C either for 20 or 45 min with constant agitation followed by trituration. Dissociated cells were spun down for 5 min at 2000 rpm and resuspended in 500 μl of resuspension media (430 μl EBSS, 50 μl albumin-ovomucoid inhibitor, 25 μl DNase). To prepare the density gradient, 600 μl of albumin-ovomucoid inhibitor was added to a 15 ml Falcon tube, and the cell suspension layered on top. Following centrifugation for 6 min at 1000 rpm, the cells were resuspended with 1x PBS/5% FBS. Dissociated cells from the retinal blocks of animal AV263 were incubated in 5 μg/ml of Alexa-488 fluorophore-conjugated PNA in 1x PBS/5% FBS for 15 min at RT before cell sorting. Sorting of PNA-positive or GFP-positive (GFP+), micro-ruby™ -positive (Ruby+) and unlabeled cells was performed on a BD FACS ARIA SORP equipped with BD FACS Diva software 8.0.1 and a 100 micron nozzle. The filters used to detect the PNA or GFP positive fraction were 505LP and 530/30BP (range 515–545 nm) off the Blue 488 nm laser. The filters used to detect micro-ruby™ were the 600LP and the 610/20BP (range 600–620 nm) off the Yellow Green 561 nm laser.

### RNA extraction and reverse transcription (cDNA synthesis)

FACS sorted cells were collected in 1x PBS/5% FBS, spun down for 5 min at 2000 rpm, and resuspended in lysis buffer. Total RNA was extracted from the cell lysate using the RNeasy mini RNA extraction kit according to the manufacturer's protocol (Qiagen, CA, Cat #74104). The RNA samples were treated with DNase (to remove genomic DNA contamination) for 30 min at 37°C followed by 10 min at 75°C to deactivate DNase. The quantity and purity of RNA were determined using a NanoDrop™ 2000 spectrophotometer (Thermo Scientific, MA) and 200 ng of total RNA used as template for reverse transcription using iScript Reverse transcription Supermix (Bio-Rad, CA, Cat #1708890) at 25°C for 5 min, followed by 42°C treatment for 30 min and then 85°C treatment for 5 min. No RT control qPCR reactions were performed with primer pairs that targeted a discrete exon to confirm that samples were free of genomic DNA contamination (data not shown).

### Quantitative real-time PCR (qPCR): primer design and validation

qPCR was performed using iTaq™ universal SYBR Green supermix (Bio-Rad, CA, Cat #172-5121) and CFX96 real-time PCR system (Bio-Rad laboratories, Inc., USA). Primers were validated in tenfold serial dilutions of sample. The cycling conditions were as follows: initial denaturation for 5 min at 95°C; 39 cycles of 15 s at 95°C, 30 s at 60°C, and a melting curve of 65°C to 95°C at an increment of 0.5°C per second. Data analyses, which included the determination of a standard curve, quantification cycle (C_q_) value, PCR efficiencies, slope of the standard curve and melt curve were automatically performed with the CFX Manager™ Software system (Version 1.6; Bio-Rad Laboratories, Inc., USA). Relative expression of five different primate retinal cell- specific genes including Rhodopsin (*RHO*; rod specific), guanine nucleotide binding protein (G protein) alpha transducin activity polypeptide 2 (*GNAT2*; cone specific), glutamate receptor Metabotropic 6 (*GRM6*; ON bipolar cell specific), glutamate-ammonia ligase/glutamine synthetase (*GLUL*; Muller cell specific), and Thy-1 cell surface antigen (*THY1*; retinal ganglion cell-specific) was evaluated. Additionally a subset of samples was evaluated with primer pairs to the different cone opsins, M/L opsin, opsin1 (cone pigments), medium, or long-wave-sensitive (*OPN1MW* or *OPN1LW*) and S opsin, opsin1 (cone pigments), short-wave-sensitive (*OPN1SW*) and other RGC expressed genes, *BRN3A*, POU class 4 homeobox (*POU4F1*), melanopsin, Opsin 4 (*OPN4*) and Retinal pigment epithelium specific protein 65 kDa (*RPE65*) each assay was carried out using 2 μl (1:10 dilution) of cDNA in a total reaction volume of 20 μl containing 500 nM of each Forward and Reverse primers. A complete list of primers, their sequences, accession number used as reference sequence and their location on the respective cDNA can be found in Supplementary Table 1. All primers were designed to be identical matches to mRNA targets of *M. fascicularis* (used in this study) and *M. mulatta* unless otherwise noted. The primer pair for M/L opsin is predicted to equally amplify both M and L opsin cDNA based on annealing location and the existing polymorphisms between the two respective genes. All samples, including the standards, and negative control were run in triplicate. PCR cycling conditions were as follows: one cycle of initial denaturation at 95°C for 5 min; 40 cycles of 30 s at 95°C, 30 s at 58°C and 1 min at 72°C and a final extension step of 72°C for 7 min. *GAPDH* was used as an internal reference since all the samples used were retina and no comparison to other tissue types were made. Relative quantification was calculated with the ΔΔCq method. Prior to transcript analysis by qPCR, validation of all primer pair was performed conforming to MIQE guidelines (Bustin et al., 2009). Specifically standard curve analysis and confirmation of target specific by melting curve analysis (single peak) was performed utilizing the whole retina from the OS eye of AV263. No signals were detected in any of the “no template” controls (Supplementary Figure 1).

## Results

### Labeling of retinal cells post-enucleation leads to insufficient sorting

**Figure 3:**
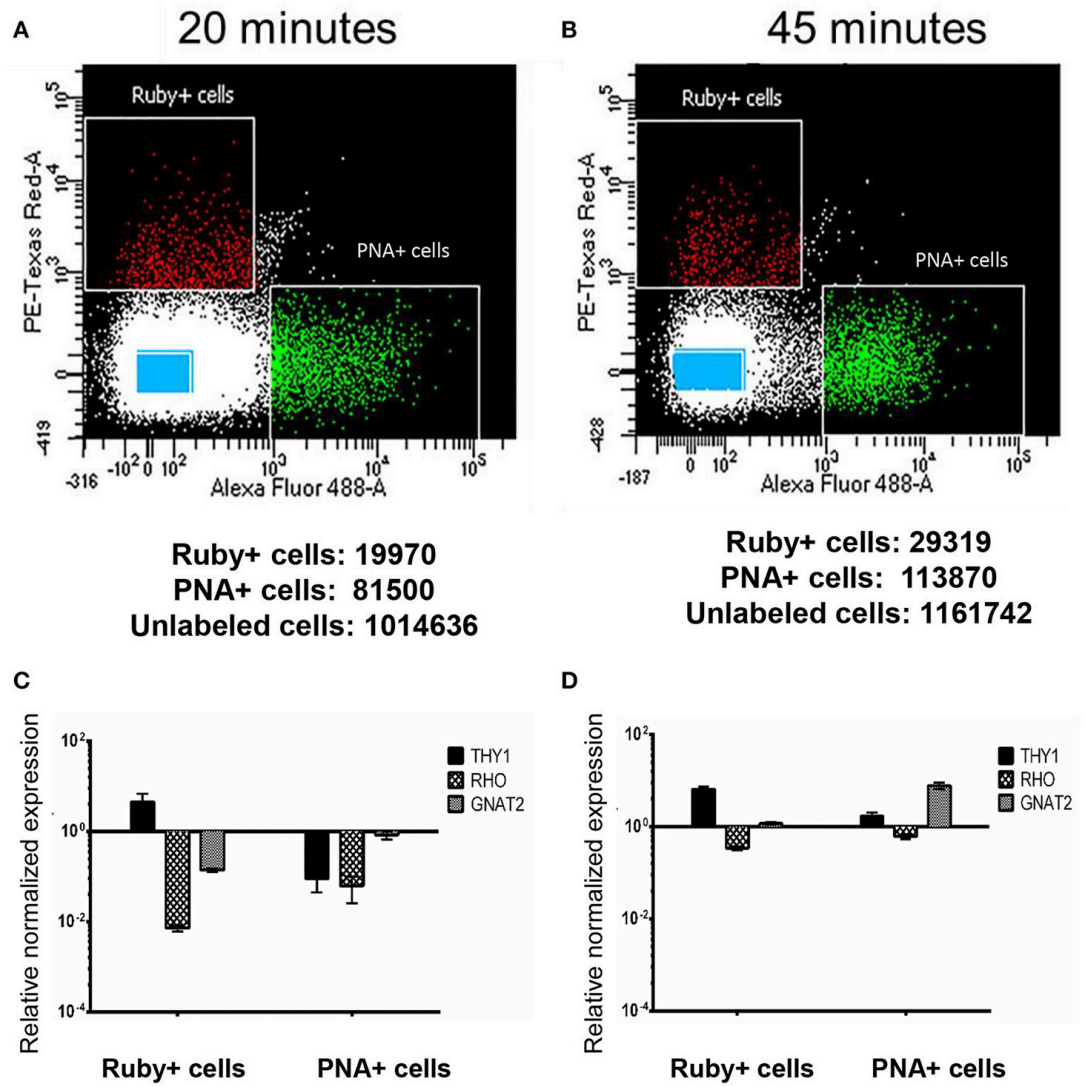
**Isolation and characterization of macaque retinal cells labeled post sacrifice**. Scatter plots show the different gating conditions used for isolating the Ruby+, PNA+, and unlabeled cells (blue rectangles) following incubation with papain for either 20 min **(A)** or 45 min **(B)**. The total numbers of sorted Ruby+, PNA+, and unlabeled cells are shown below each respective scatter plot. Expression of *THY1, RHO*, and *GNAT2* in Ruby+ and PNA+ cells following 20 min **(C)** or 45 min **(D)** papain dissociation. Expression was normalized to *GAPDH* and is shown relative to expression in unlabeled cells. Error bars represent SEM.

Retina samples from animal AV263 were subjected to two different papain concentrations and incubation times that were selected based on conditions used previously by Han et al. in primate retina (Han et al., 2000). The dissociated samples then underwent FACS to isolate Ruby+ positive (561 nm), PNA+ (488 nm) and unlabeled cells. The scatter plots and the total number of micro-ruby™, PNA positive and un-labeled cells collected under both conditions is presented in Figure 3. Regardless of the method used (20 vs. 45 min incubation in papain), some separation was achieved based on the differential profile of gene expression of *THY1, RHO*, and *GNAT2* between the collected Ruby+, PNA+, and unlabeled cell populations (Figure 3). Ruby+ positive cells were enriched for *THY1* expression regardless of the initial duration of papain incubation but *GNAT2* expression was only enriched in PNA positive cells when retina was dissociated for 45 min. Based on these preliminary results, we selected 45 min as the optimal duration with which to dissociate macaque retina in papain prior to sorting. However, due to the overlap of *THY1* and *GNAT2* in the PNA+ and Ruby+ captured cells relative to captured un-labeled cells, we concluded that labeling post-enucleation was insufficient to effectively isolate cones from RGCs. Additionally, PNA labeling only enables capture of cone photoreceptors. We therefore chose to utilize strategies that labeled RGCs and all photoreceptors in-life.

### Subretinal AAV5-hGRK1-GFP and LGN injections of micro-ruby efficiently and selectively label primate photoreceptors and RGCs, respectively

**Figure 4:**
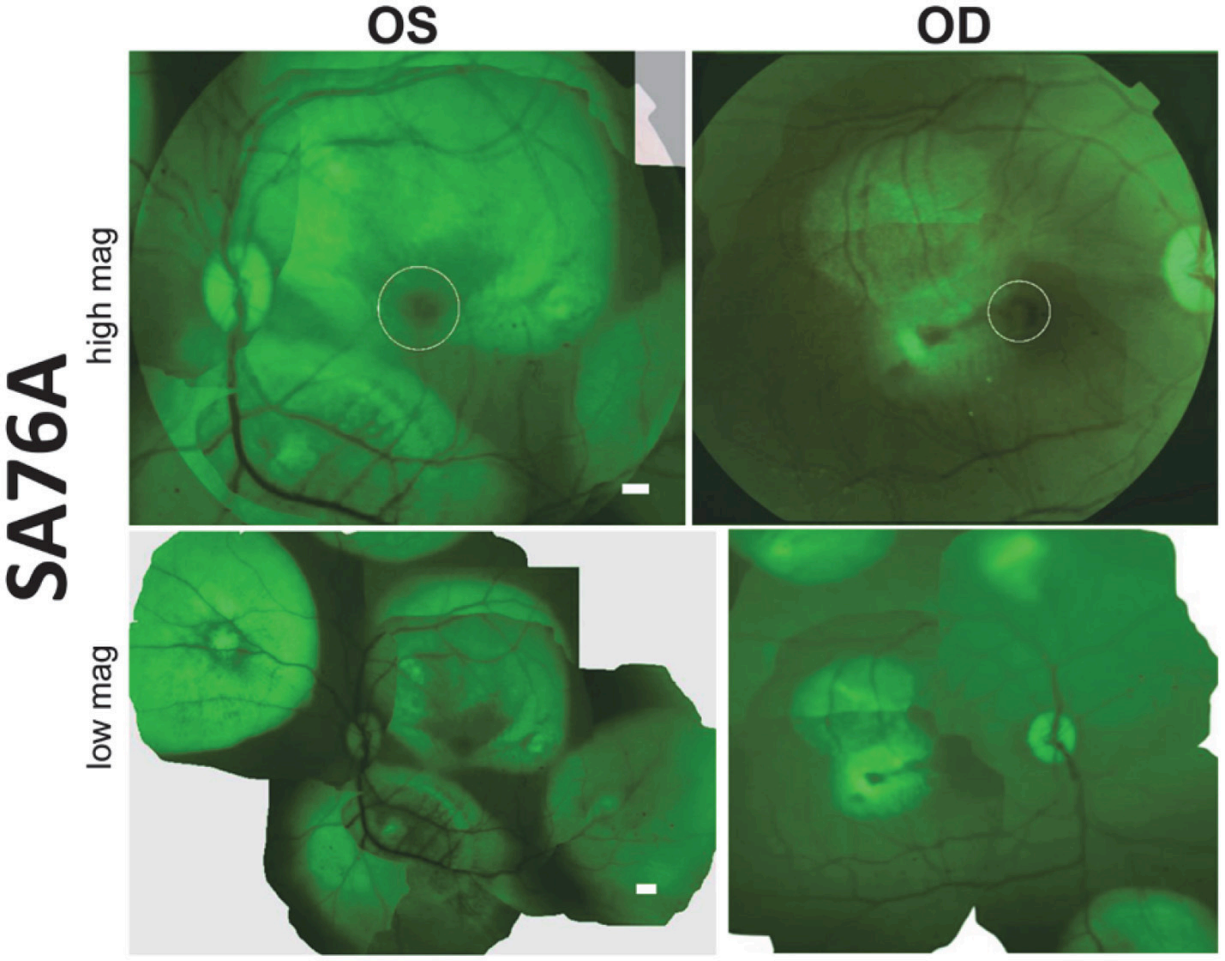
**Fluorescent fundus images taken 20 days post- subretinal injection with AAV5-hGRK1-GFP in SA76A. Upper panel:** 50° field of view images reveal green fluorescent protein (GFP) fluorescence and the location of the macula/fovea in both eyes (white circles). **Lower panel:** Montages show the location of the five injection blebs placed in each eye. Scale bars = 500 μm.

Subretinal delivery of AAV5-GRK1-GFP has previously been shown to drive GFP expression exclusively in rod and cone photoreceptors of all regions of macaque retina (Boye et al., 2012). Utilizing this same AAV vector, we performed a series of subretinal injections in both eyes of animal SA76A with the goal of transducing photoreceptors over a large retinal area. Prior to sacrifice, fluorescence fundus imaging was performed to assess GFP expression. En face fluorescent images revealed robust GFP expression at 20 days post-injection in both the central and peripheral retina that was restricted to the area of each respective subretinal bleb. GFP expression was observed in the macular blebs of each eye however its intensity was decreased relative to that seen in the peripheral blebs, an observation possibly due to natural pigmentation of the macular retina (Figure 4). Notably, by restricting individual bleb volumes to <100 μl, we were able to place at least 5 separate subretinal blebs in different regions of macaque retina, including the macula. Based on clinical observations and fundus images, there was no apparent damage to the retina.

**Figure 5:**
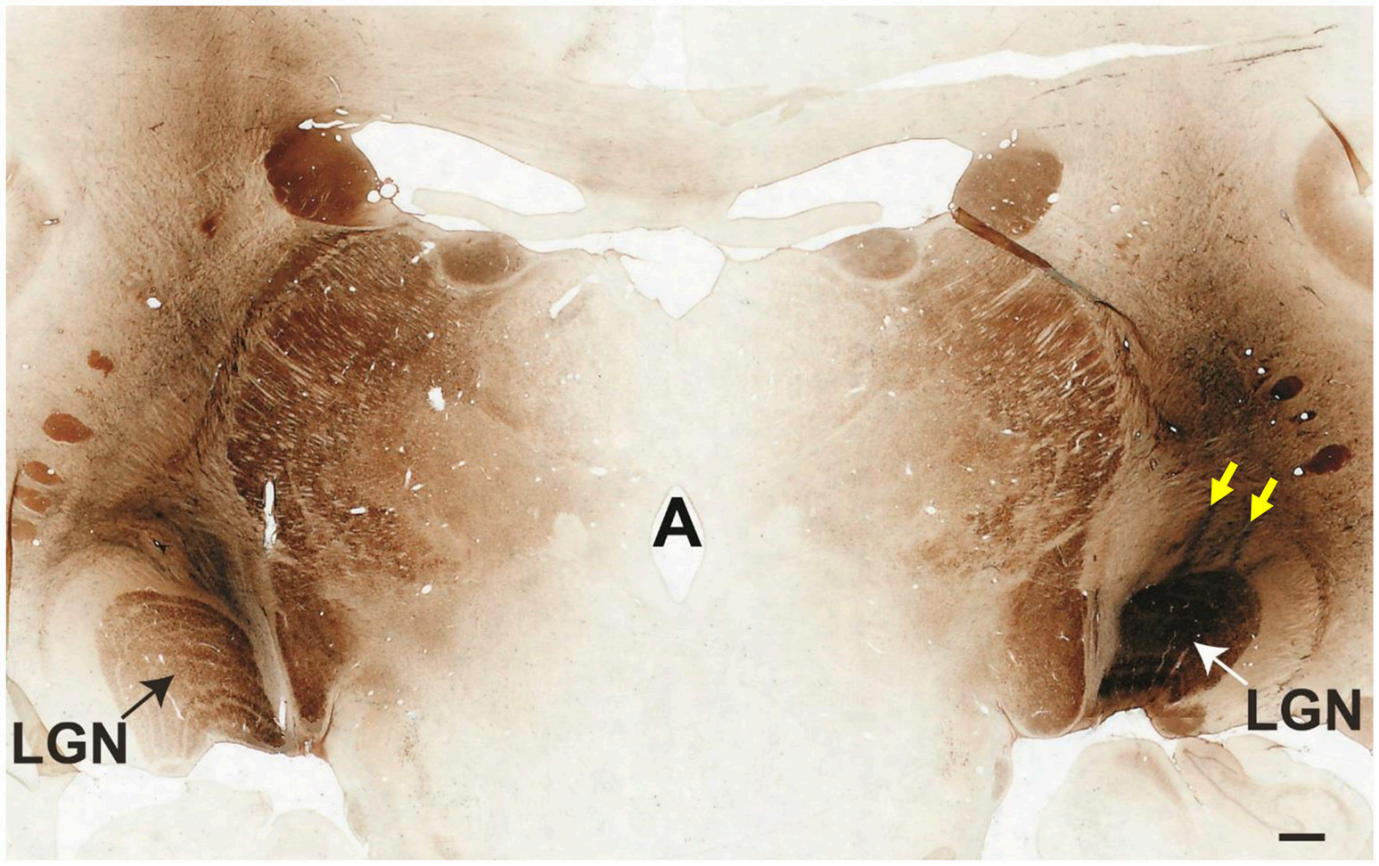
**Coronal cross-section through the thalamus showing the locations of bilateral micro-ruby™ injections into the LGN**. The left medial LGN is clearly labeled, as is the adjacent optic tract. The entire right LGN is labeled. Yellow arrows denote two injection tracks that targeted the nucleus. Scale bar = 1 mm.

**Figure 6:**
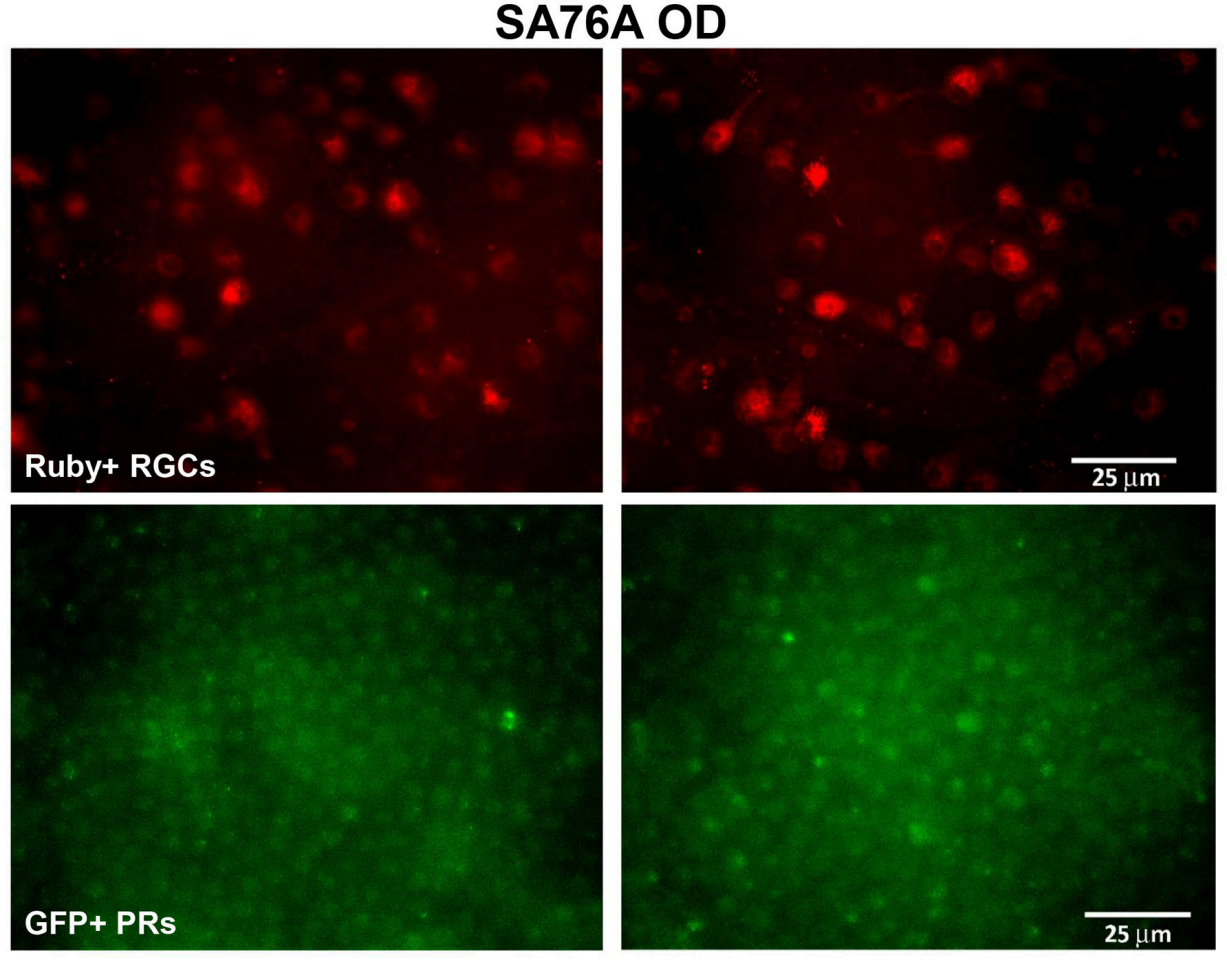
**Representative fluorescent images of retinal flat mounts from the inferior temporal portion of SA76A's OD eye**. The **upper panel** shows micro-ruby™ labeled retinal ganglion cells and the **lower panel** shows AAV5-hGRK1- mediated GFP expression in photoreceptors. RGC, retinal ganglion cells. Scale bar = 25 μm.

Electrophysiologically-guided injections of the LGN were successful in labeling substantial regions of the LGN and many fibers of passage in the adjacent optic tract. Coronal brain sections through LGN confirmed successful, bilateral micro-ruby™ delivery (Figure 5). Labeling was most intense in the right LGN with horseradish peroxidase (HRP)-stained injection tracks easily visualized (yellow arrows, Figure 5). AAV5-hGRK1-GFP mediated GFP expression in photoreceptors and micro-ruby™ labeling in RGCs were also verified post mortem by direct visualization of cells in a retinal flat mount from the inferior temporal portion of SA76A's OD eye (Figure 6).

### Ruby+ and GFP+ cell populations can be isolated by FACS

**Figure 7:**
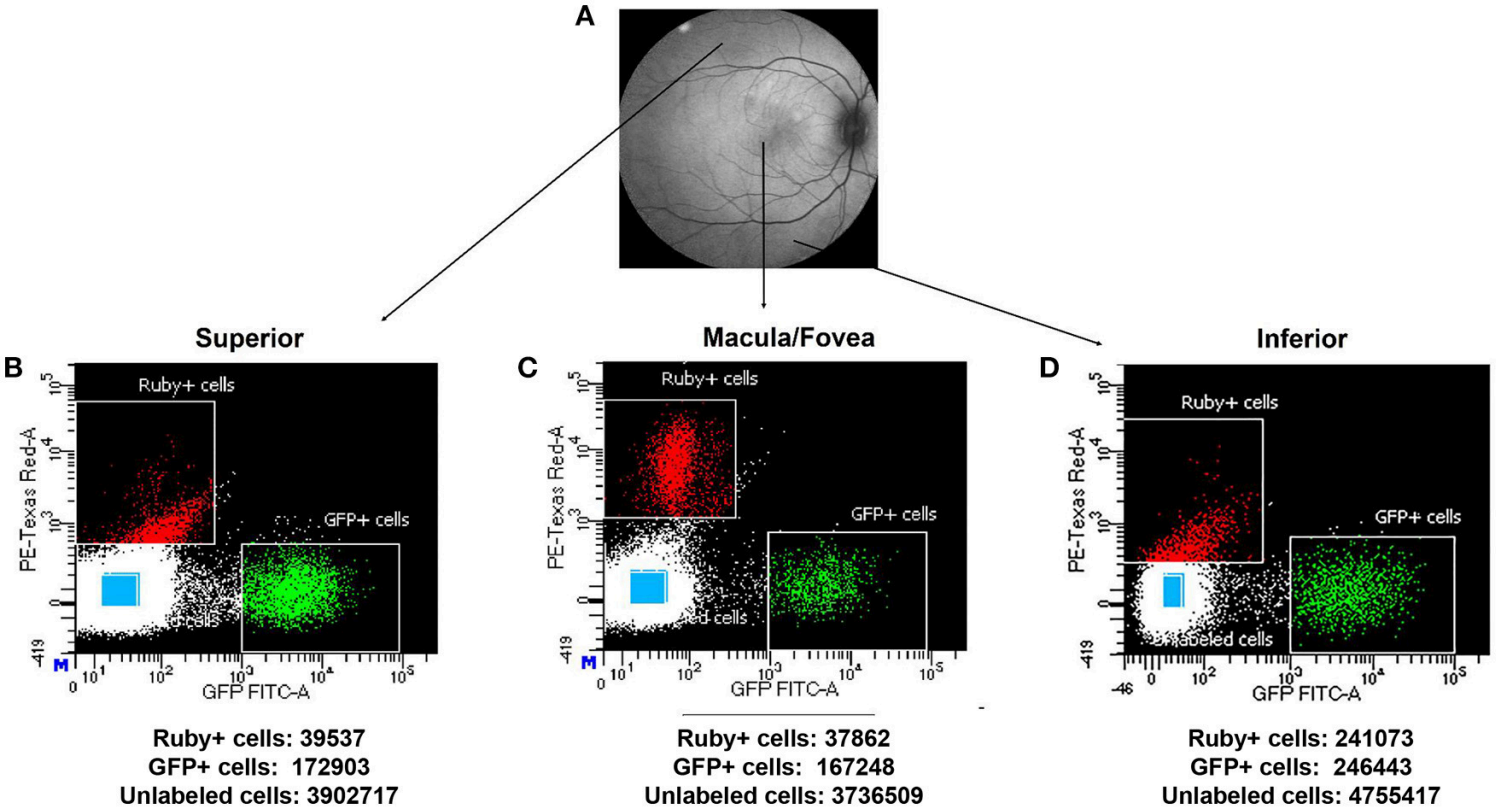
**Representative macaque fundus image is shown in (A)** to denote retinal locations from which dissociated cells were used for FACS analysis. Scatter plots showing the different gates used for isolating the Ruby+, GFP+, and unlabeled cells (blue rectangles) sorted from superior **(B)**, macula/fovea **(C)**, and inferior **(D)** retinal regions. The total number of sorted Ruby+, GFP+, and unlabeled cells isolated by FACS is shown beneath each respective scatter plot.

Using the optimal dissociation condition described above, GFP positive cells (putative photoreceptors), micro-ruby™ positive cells (putative RGCs) and unlabeled cells from macular, superior and inferior regions of SA76A's retina were isolated using FACS. Scatter plots and the total numbers of cells captured from each population and region are presented in Figure 7. The GFP+ population within all anatomical regions was easily identifiable on scatterplots as an isolated cluster. In contrast, more efficient clustering of Ruby+ cells was observed in the macula/fovea relative to the inferior and superior region samples.

### Analysis of gene expression in sorted cell populations validates enrichment for RGC and photoreceptors

The captured populations of Ruby+, GFP+, and unlabeled cells were first analyzed for expression of RPE65 to confirm that manual separation of neural retina from the underlying RPE efficiently removed the latter cell type from samples. *RPE65* expression in the unlabeled cell populations was ~3 log levels lower than that seen in whole retina and was below the level of detection in the Ruby+ and GFP+ cell samples (data not shown).

**Figure 8:**
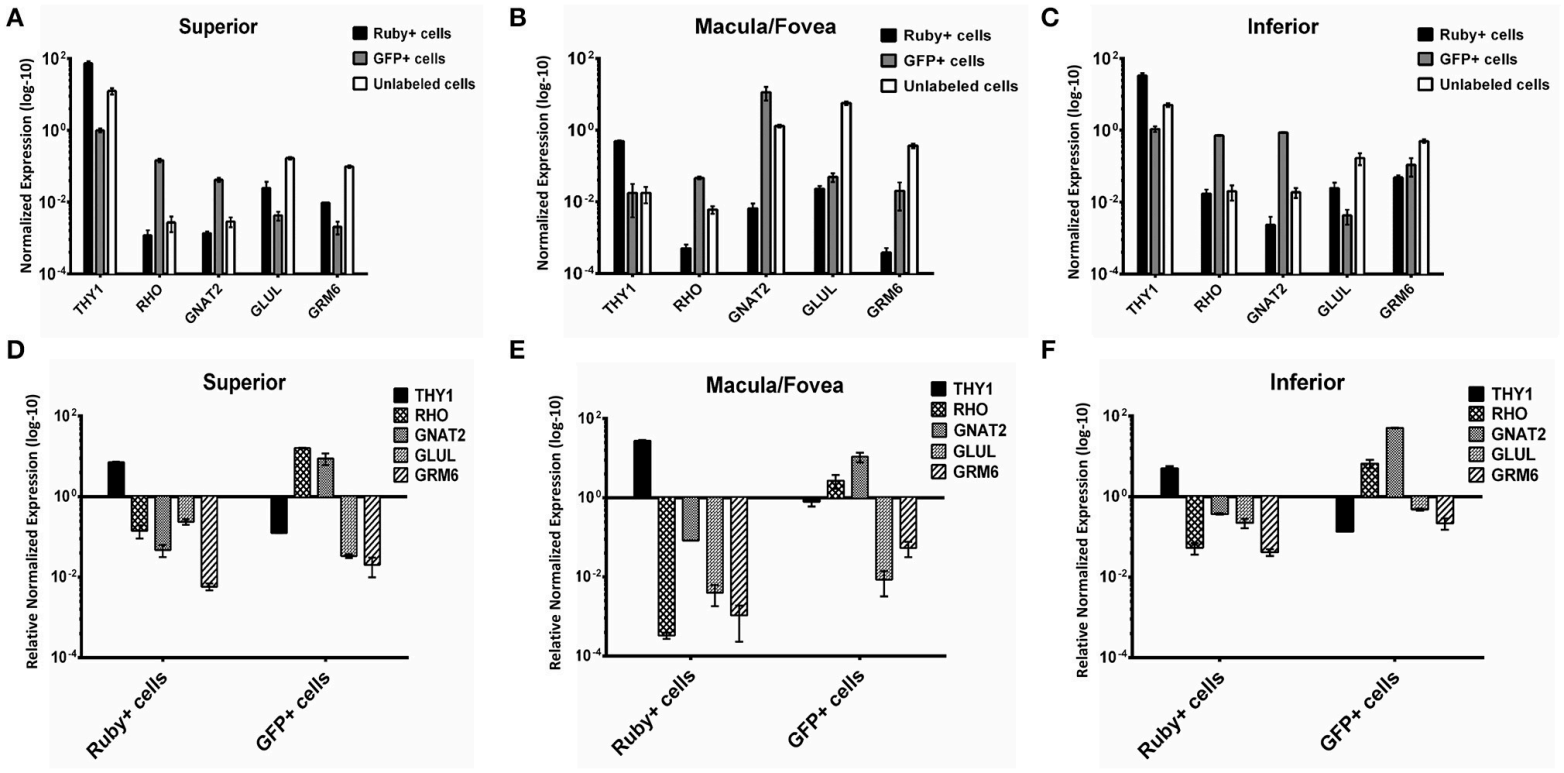
**Expression of ***THY1***, ***RHO***, ***GNAT2***, ***GLUL***, and ***GRM6*** in Ruby+, GFP+, and unlabeled cells sorted from superior (A)**, macula/fovea **(B)**, and inferior **(C)** retina. Expression was normalized to *GAPDH*. Expression of *THY1, RHO, GNAT2, GLUL*, and *GRM6* in Ruby+ and GFP+ populations relative to the unlabeled cell populations from superior **(D)**, macula/fovea **(E)**, and inferior **(F)** retina. Expression was normalized to *GAPDH* and is shown as relative to anatomically matched unlabeled cell population. Error bars represent SEM.

A comparison of the expression levels of selected retinal genes (*THY1, RHO, GNAT2, GLUL*, and *GRM6*) normalized to *GAPDH* in the Ruby+ and GFP+ cell populations showed a pattern consistent with enrichment for RGCs or photoreceptors, respectively in each of the anatomical regions sampled (Figure 8). For example, in the macula/fovea sample, *THY1* expression was highest in the Ruby+ cells while *RHO* and *GNAT2* expression were highest in the GFP+ cells. In addition, expression of all non-RGC- and non-photoreceptor- specific genes were highest in the unlabeled cell populations. When gene expression within each respective fluorescent cell population was normalized to levels observed in the unlabeled cells from that same anatomical region, the magnitude of enrichment was even more apparent (Figure 8). The enrichment factors (fold increase or decrease) for each gene in the Ruby+ and GFP+ cell populations from each anatomical region are presented in Table 3.

**Table 3:** **Fold enrichment of various genes in Ruby+ and GFP+ cell populations relative to un-labeled cells from same region**.

	**Superior**		**Macula/Fovea**		**Inferior**	
**Target**	**Ruby+ population**	**GFP+ population**	**Ruby+ population**	**GFP+ population**	**Ruby+ population**	**GFP+ population**
*THY1*	7.21	0.126	27.813	0.79	5.063	0.135
*RHO*	0.142	16.175	0.0003	2.722	0.053	6.68
*GNAT2*	0.047	8.917	0.083	10.874	0.37	51.268
*GLUL*	0.238	0.034	0.004	0.008	0.223	0.48
*GRM6*	0.005	0.02	0.083	10.874	0.041	0.218

### Cone opsin expression in GFP+ populations

**Figure 9:**
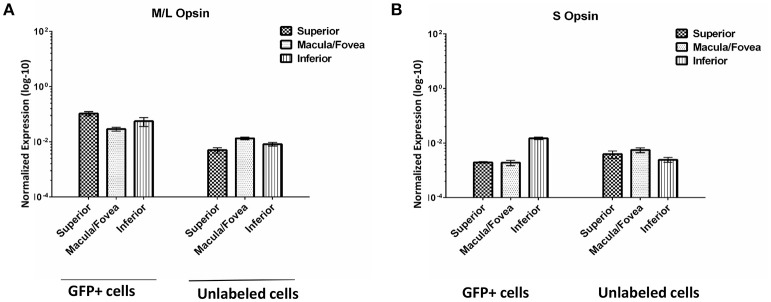
**Expression of M/L Opsin (***OPN1LW***) (A)** and S Opsin (*OPN1SW*) **(B)** in GFP+ and unlabeled cells sorted from superior, macula/fovea, and inferior retinal regions. Expression was normalized to *GAPDH*. Error bars represent SEM.

We evaluated the expression of M/L opsin and S opsin in GFP+ cell populations sorted from macula/fovea, superior and inferior regions as well as in the corresponding unlabeled cells from the same regions (Figure 9). As expected, opsin expression was much higher in the GFP+ cell populations than in unlabeled cells. Based on the distribution of cone subclasses in the primate retina, it was anticipated that M/L opsin would be enriched in the GFP+ cells from the macula/fovea relative to those isolated from the superior and inferior retina, as was seen for the unsorted cell populations (Curcio et al., 1987). However, in GFP+ cells isolated from macular retina, M/L opsin expression was slightly lower than that seen in GFP+ cells from superior or inferior retina (Figure 9). We believe this result is due either to a lower recovery efficiency of GFP+ cells in the macula/fovea relative to peripheral retina and/or inefficient gating conditions in the macular sample due to small cone size. M/L opsin expression in GFP+ cells from the superior retina was higher than that seen in the inferior sample (Figure 9). Increased M/L opsin expression in superior relative to inferior retina is consistent with the gradient distribution of this cone subclass in the macaque retina (Wikler and Rakic, 1990). S-opsin expression was higher in GFP+ cells of the inferior retina relative to that seen in superior and macula/fovea samples (Figure 9).

### Expression of RGC associated genes in ruby+ cells

A comparison of *OPN4* expression in Ruby+ RGCs revealed modest enrichment in the macula/fovea and inferior retina relative to that seen in the superior. In contrast, *BRN3A* expression was clearly enriched in Ruby+ cells from superior retina relative to that seen in macula/fovea and inferior samples (Supplementary Figure 2).

## Discussion

In this study, we compared methods for creating sortable photoreceptors and RGCs in macaque, and determined suitable conditions for retinal dissociation and FACS to isolate these cells from a mixed population. Our results show that a post mortem approach wherein labeling of RGCs and photoreceptors was achieved via incubation of the optic nerve head in micro-ruby™ and PNA staining of dissociated neural retina, respectively, failed to promote truly efficient separation of these cell populations. RGC specific transcript was enriched within the PNA positive cell population, indicating that sorted retinal cells labeled via this method lacked sufficient purity. However, this initial experiment was useful for determining suitable parameters for papain dissociation of retinal tissue. We next investigated an *in vivo* approach built upon our previous work establishing that photoreceptors (rods and cones) can be specifically labeled by subretinally- delivered AAV5-hGRK1-GFP (Boye et al., 2012) and that RGCs can be specifically labeled via LGN tracer injections (Dacey et al., 2003, 2005). The bimodal, *in vivo* approach reported here resulted in isolation of GFP+ and Ruby+ cell populations from macaque retina that, based on their gene expression profiles, were heavily enriched for photoreceptors and RGCs, respectively.

We noted that the enrichment pattern of retinal cell specific genes agreed with the known spatial distribution of their respective cell types. For example, *GNAT2* was heavily enriched in GFP+ cells within the macula/fovea. Conversely, there was relatively higher *RHO* expression in the peripheral samples. Both results align well with the known distribution of rods and cones within primate retina (Snodderly et al., 1984a,b). Additionally, in the unlabeled cell populations *GLUL* was most prevalent in the Macula/fovea sample, consistent with the increased density of Müller cells around the primate fovea (Distler and Dreher, 1996).

Labeling of the putative photoreceptor population was dependent on successful delivery of AAV vector and expression of the GFP transgene. In life fluorescent imaging and FACS, as well as our previous characterization of this vector (Boye et al., 2012) suggest that this was successful. Within the 4 mm foveal/macular punch, we isolated ~167,000 GFP+ cells. Based on prior work by Curcio and colleagues, an estimate of the total number of photoreceptors for this area is 500,000 cones and 5,000,000 rods, which suggests a 3% recovery rate. This rate can be significantly improved by adjusting the gating size and intensity settings to accept rods and foveal cones. Our finding that M/L and S opsin expression levels were lower in GFP+ cells isolated from macula/fovea relative to peripheral retina, suggests that the efficiency of cone recovery for this sample was low. One possible explanation for this is that delivery of AAV5-hGRK1-GFP to cones or expression in the cones within the macular bleb was less efficient than that achieved in the periphery. While in life fundus images seem to support this (the central retina appears darker than the periphery), this is a typical observation due to macular pigment in this region and does not necessarily denote a reduction in AAV-mediated GFP expression (Wikler and Rakic, 1990). The maximum absorbance of these pigments is 460 nm (Snodderly et al., 1984a,b) which interferes with the 488 nm excitation light resulting in a reduced fluorescence intensity from transduced cells in this region. Still, we did note that fluorescence appeared lower in the macular blebs of animal SA76A than that observed in our previous study utilizing the same AAV5-hGRK1-GFP vector preparation at the identical concentration (Boye et al., 2012). Notably, no vitrectomy was performed prior to subretinal injection of vector in the current study whereas macaques previously described received at least a core vitrectomy (Boye et al., 2012). It is possible that the lack of any vitrectomy in animal SA76A reduced the efficiency of macular detachment and thus transduction of cones. It is also possible that cone photoreceptors degenerated as a consequence of being transduced by the AAV5-hGRK1-GFP vector as was observed following subretinal delivery to the canine retina (Beltran et al., 2010). However, in the canine experiments, the concentration of vector resulting in cone loss was substantially higher (1.5 × 10^13^ vg/ml) than what was used in this study (1.0 × 10^12^ vg/ml). Furthermore, there was no evidence of cone degeneration in our previous study wherein 1.0 × 10^12^ vg/ml was delivered subretinally to NHP (Boye et al., 2012). It is also worth noting that in pre-clinical dose escalation studies of AAV-RPE65 vector tested in dog and macaque, dogs were found to be more sensitive to AAV mediated retinal toxicity (Jacobson et al., 2006a,b).

An alternative explanation for the lower than anticipated recovery of cones is that the restrictive gating conditions used to isolate GFP+ cells was insufficient to capture macular cones. The small size of foveal cones relative to those in the periphery likely reduced their overall GFP fluorescence intensity and thus, when using gating conditions suited for cells with higher fluorescence, a percentage of GFP+ foveal cones was lost. It is apparent in the scatter plot that there are a significant number of cells expressing lower levels of GFP that fell outside of the capture (Figure 7B). It is possible these cells are the small M/L foveal cones. Notably, expression of cone specific genes, M/L opsin, S opsin, and *GNAT2* within the unlabeled macular cells was much lower than that seen in the GFP+ population collected within this region suggesting that the “missing” macular cones were not in the unlabeled cell population captured by FACS as would be expected if they were lacking any GFP labeling.

Good separation of Ruby+ RGCs was achieved in all retinal regions but was most pronounced in the macula/fovea sample (Figure 7). This we attribute, in part, to the high RGC density in the macula (Dacey, 1994; Dhingra et al., 2008). However, *THY1* expression was not highest in the macula/fovea samples relative to the other anatomical regions, either in the putative RGC population or the unlabeled sample. The reason for this is unknown. Previous work has shown that melanopsin-containing RGCs are at a higher density centrally in the macaque retina (Dacey et al., 2005; Liao et al., 2016). While we found that *OPN4* expression was enriched in the macula/fovea relative to the superior retina, expression was similar to that observed in the inferior sample (Supplementary Figure 2).

While these results were primarily generated using one animal, we designed the study such that there were multiple experimental replicates. For example, AAV5-hGRK1-GFP was subretinally injected into both eyes. Within each of those eyes, multiple subretinal injection blebs were created, in all cases successfully. Retinas were subdivided into 3 anatomical regions that were sorted and analyzed separately. In each of those sorting experiments, results of the analysis consistently showed strong enrichment for each respective cell type. The only portion of the study that was not replicated was the LGN injections. Previously, we demonstrated this procedure to be highly reproducible in several studies utilizing multiple macaques (Dacey et al., 2003, 2005). Given that RGCs are the only retinal cell type to innervate the optic tract, there was no-concern regarding cell-type specificity of micro-ruby dye incorporation. Taken together, our previously published work and results presented herein support the conclusion that this methodology is a reliable approach for creating sortable photoreceptors and RGCs in macaque.

A major utility of a method to selectively recover different retinal cell types is for use in the screening or characterization of novel reagents for their ability to target these cell types. The AAV vector toolkit is rapidly expanding with novel capsid variants emanating from both rational design and directed evolution techniques (Wu et al., 2006; Vandenberghe and Auricchio, 2012). In addition, novel cellular promoters are being identified to target AAV-mediated transgene expression to specific retinal cells thereby increasing the safety of clinical candidate vectors (Ye et al., 2016). While identification of capsid motifs and cellular promoters that promote photoreceptor- or RGC-specific targeting may be studied in mouse, clinical translatability is best assessed by validation in primates, which possess foveas. Novel gene delivery vectors can be engineered to contain “barcodes” and the selective capture of a tissue or cell type of interest followed by next generation sequencing of the recovered nucleic acids can generate a frequency distribution (Adachi et al., 2014; Marsic et al., 2015). Importantly, this can be done within an individual animal allowing simultaneous comparison of a multitude of different reagents. It is possible that the transduction efficiency of novel vectors and/or activity of novel promoters will be different in diseased vs. healthy retina. Notably, this method may also be applied to other non-primate species such as canine. A variety of canine models of inherited retinal disease exist and thus afford the opportunity to interrogate this phenomenon. Taken together, the methods developed and described in this report will provide a basis for utilizing these emerging technologies to more rapidly identify and characterize gene delivery reagents capable of targeting primate RGCs and photoreceptors.

## Author contributions

All authors listed, have made substantial, direct, and intellectual contribution to the work, and approved it for publication.

### Conflict of interest statement

The authors declare that the research was conducted in the absence of any commercial or financial relationships that could be construed as a potential conflict of interest. Despite hosting the research topic together with one of the authors of this manuscript, the handling Editor states that the process met the standards of a fair and objective review.
