# Supplementary figures and images for: Novel Methodology for Creating Macaque Retinas with Sortable Photoreceptors and Ganglion Cells
Source: Front Neurosci. 2016 Dec 1;10:551. doi: 10.3389/fnins.2016.00551 (PMC5131003; doi:10.3389/fnins.2016.00551)

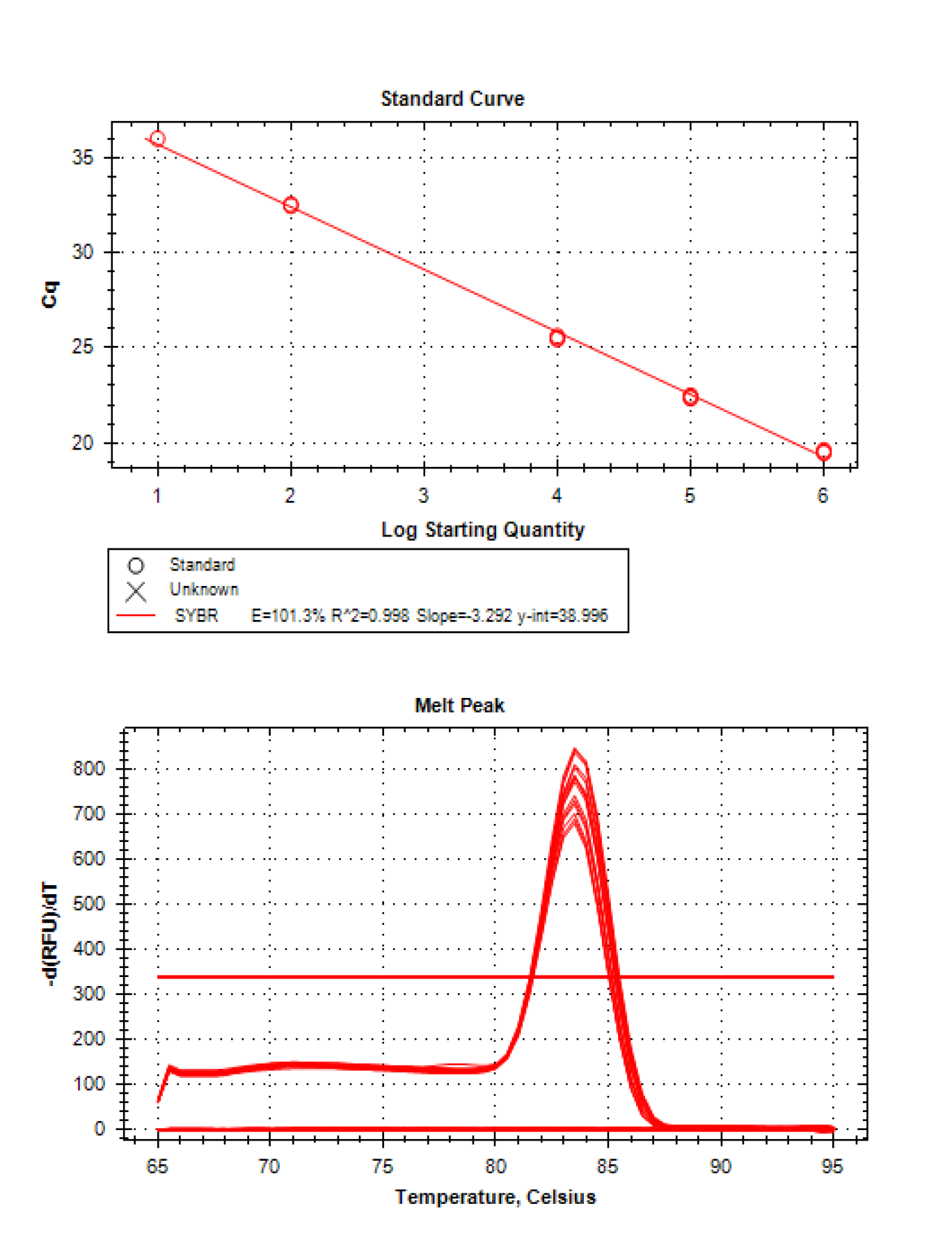

Supplement: Supplementary file 2 [file Image1.TIF]

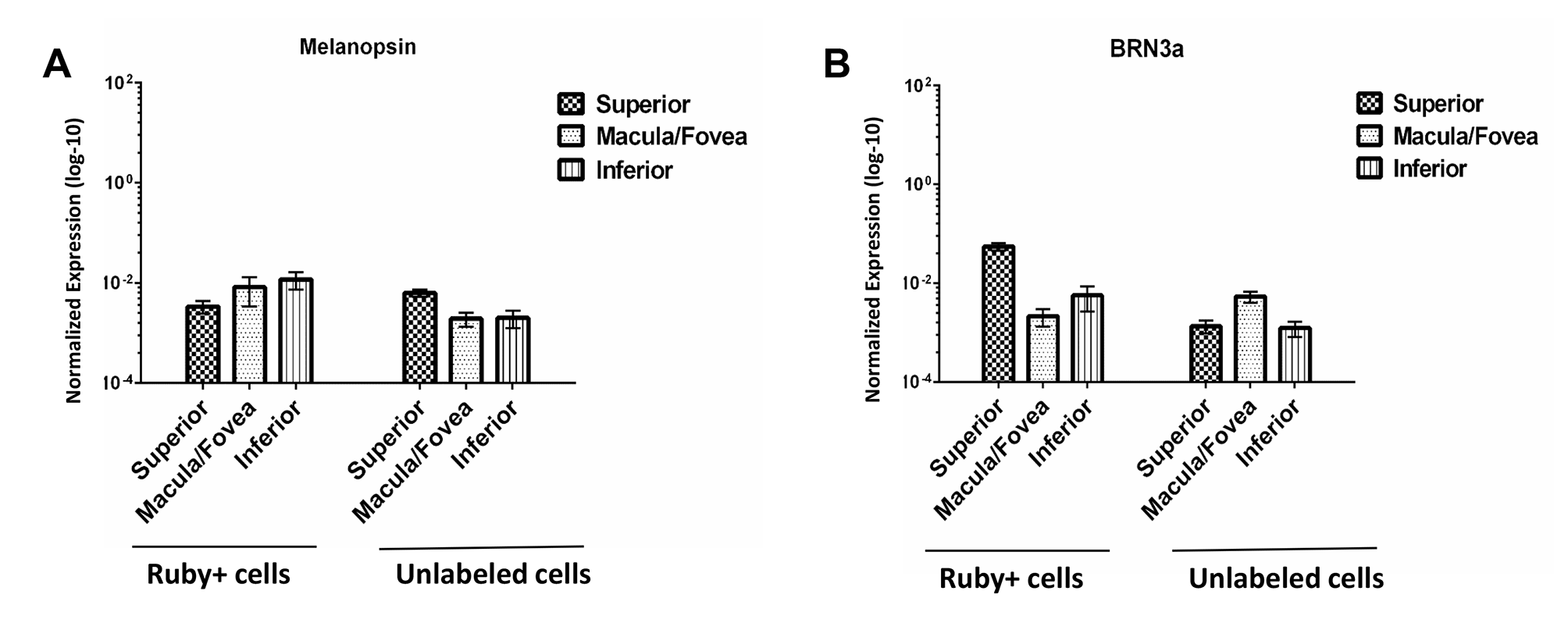

Supplement: Supplementary file 3 [file Image2.TIF]
